# Dr. Jacinto Convit (1913–2014)

**DOI:** 10.4269/ajtmh.14-0316

**Published:** 2014-08-06

**Authors:** Alberto E. Paniz Mondolfi, Barry R. Bloom

**Affiliations:** Department of Laboratory Medicine; Yale University School of Medicine; New Haven, Connecticut; and; Instituto de Biomedicina; Laboratorio de Bioquímica/Fundación; Jacinto Convit Caracas, Venezuela; Harvard School of Public Health; Department of Immunology and Infectious; Diseases and the Department of Global; Health and Population; Boston, Massachusetts

On May 12, 2014, Jacinto Convit, MD, died in Caracas, Venezuela at the age of 100. Dr. Convit was a major figure in the history of leprosy and parasitology research. Although best known for his fundamental and lasting contributions to the study of *Mycobacterium leprae*, the causative agent of leprosy (Hansen's disease), his impact on research into many parasitic agents and the field of immunotherapy was significant.

**Figure 1.**
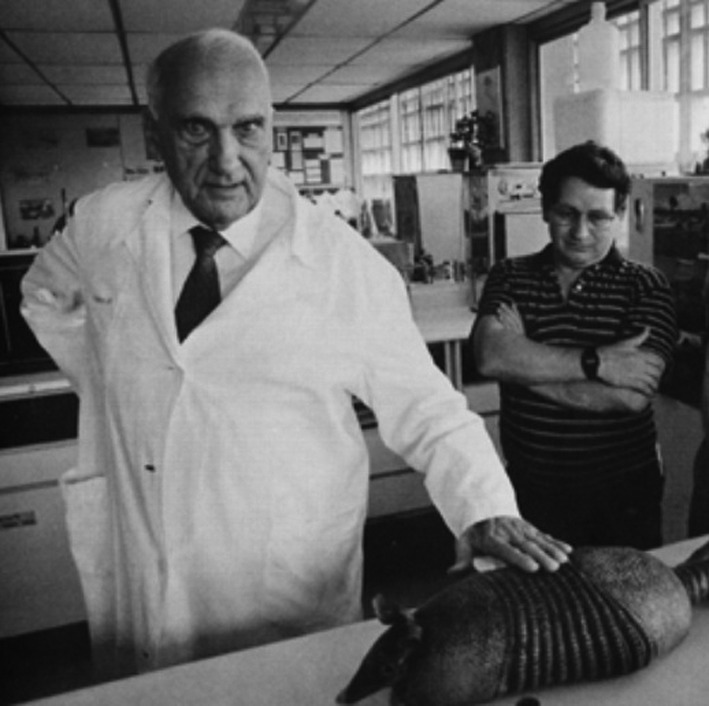
Dr. Jacinto Convit in his Laboratory at the Institute of Biomedicine in Caracas, Venezuela handling a nine-banded armadillo, the animal model used to obtain large quantities of the uncultivable leprosy bacillus for vaccine development (Image: Jacinto Convit Foundation).

Dr. Convit's professional career covered a period of over 70 years. It began in 1938 when he received his doctorate of medicine degree from the Universidad Central de Venezuela in Caracas. At that time his home country of Venezuela was a rural, poor, and disease-ridden country in which any person suspected of suffering from Hansen's disease was stigmatized and condemned to isolation. The conditions under which these patients were confined at Leprosaria, together with Convit's extraordinary human sensitivity, played a key role in his lifelong crusade against stigmatization of leprosy patients. At age 23, in the era where no cure was available for this feared disease, Convit (Figure 1) decided to devote his life to developing a cure for these patients and decided to move into one of the largest leprosaria of Venezuela, the Leprosy Clinic of “Cabo Blanco.” His first goal was to end prejudice and exclusion condemning this disease, an objective that he achieved soon after taking over the National Directorate of Leprocomiums, when he convinced the government to free the patients from isolation. His work helped change the leprosy control measures in Venezuela, replacing compulsory isolation with ambulatory treatment.

**Figure 2.**
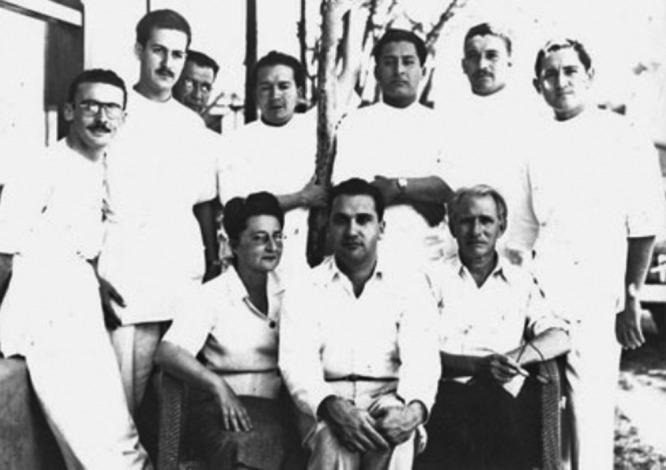
Dr. Jacinto Convit and collaborators at the Leprosy Clinic of “Cabo Blanco” (Image: Jacinto Convit Foundation).

During these tough years, Dr. Convit also volunteered as a physician in other leprosy clinics around the country and devoted a great amount of time to study the diverse clinical and epidemiological aspects of leprosy. He managed to inspire and form a small team of medical students, pharmacists, and nurses that devoted day and night to seeking a cure for patients (Figure 2). At first, the only resource available was Chaulmoogra oil, a poorly effective option that had severe nauseating effects when taken orally and which was very painful when administered intramuscularly or as subcutaneous injections. This led Convit and his team racing to find a suitable therapeutic option, a goal that would soon be achieved after proving the efficacy of sulfones and several of its derivatives on leprosy in the early 1940s.

One of Convit's major achievements was the creation of regional public health dermatology services (PHDSs) throughout the country, which allowed him not only to implement ambulatory treatment of leprosy patients, but also to provide health education and the control of contacts. These regional services progressively expanded their range of activities to include other endemic diseases such as leishmaniasis and onchocerciasis and were replicated as models and integrated into national health programs of several other countries in Latin America.

In 1960, the World Health Organization (WHO) commissioned Dr. Convit to implement a drug development and surveillance program for the treatment of several parasitic diseases, and to lead the Cooperative Center for Drug Evaluation in the Americas. Later in 1971, he was appointed by WHO as the Director of the Cooperative Committee for the Study and Histologic Classification of Leprosy, and in 1973 was appointed Director of the Pan American Center for Research and Training in Leprosy and Tropical Diseases (WHO/PAHO). In addition, he was also appointed as a Member of the WHO panel of experts who helped draft the Report of the Expert Committee in 1962–1967 and 1972.

Dr. Convit was also renowned for pioneering in the field of immunotherapy by developing in the mid-seventies a successful model of immunotherapy for leprosy combining purified leprosy bacilli obtained from the nine-banded armadillo (Figure 2) and the powerful cell-mediated immunity Bacillus Calmette-Guérin (BCG) vaccine as an adjuvant. Such a model was also applied to the treatment of leishmaniasis, an approach that has helped thousands of patients have access to therapy during shortages of antimonials and with a far less cost and comparable efficacy.

Jacinto Convit's academic career was extraordinary. He began his teaching career in the Department of Tropical Medicine at the Universidad Central de Venezuela, and later joined as a full-time Professor at the Department of Dermatology of this same University. In 1976 he founded the fellowship training program in Dermatopathology and in 2000 he founded the Masters course in Epidemiology of Endemic Diseases. He also contributed to the creation of the National Institute of Dermatology, and in 1984 founded the Institute of Biomedicine, a state-of-the-art research institution devoted to basic science investigations and public medical service for dermatological and other tropical diseases. He pursued additional training in Dermatology, Epidemiology, and Biostatistics at Columbia, Case Western Reserve and Stanford University and was a visiting Professor at Stanford and the University of Miami. He published more than 300 original articles.

As a skillful clinician with a superb clinical insight, he contributed greatly to the study and classification of a plethora of dermatological illnesses. He was the first person to describe the malignant pole of American cutaneous leishmaniasis naming it “anergic” or “diffuse cutaneous leishmaniasis” and also was included among the authors who first described the rare and peculiar pigmentary disorder “Erythema Dyschromicum Perstans (ashy dermatosis).” However, his contribution to medicine and infectious diseases was far wider than his particular interest in leprosy and leishmaniasis. He studied, wrote, and lectured extensively on many endemic diseases such as systemic and subcutaneous mycoses, lymphatic filariasis, onchocercosis, and intestinal parasitosis. Even in his final years, Dr. Convit asked all of his collaborators and friends to carry one last and ambitious project, to extrapolate his lifelong experience in microbial immunotherapy models to the cancer world. “There is a lot we have to learn about immunity and cancer, and bugs may be holding a clue for progress in this field,” he once stated in his final years.

Dr. Convit's prominence within the field of Tropical Medicine was acknowledged by a series of highly prestigious awards, including the Prince of Asturias Prize in science and technology, France's Legion of Honor, The Medal “Health for All” awarded by the World Health Organization (WHO), the Abraham Horwitz Prize for leadership in Inter-American Health, the Armand Frappier Medal, and the Alfred Soper Award and “Hero of Health” medal awarded by the Pan American Health Organization. He was also included among the 65 Caring Physicians of the World by the World Medical Association.

Over the years, he held a number of academic posts at different institutions and chaired at numerous international meetings. He was elected president of the International Leprosy Association, which he led for a decade (1968–1978); and was honorary member of the American Society of Tropical Medicine and Hygiene, the Royal Society of Tropical Medicine and Hygiene, the American Dermatological Association, the Society for Investigative Dermatology, and a founding member of the International Society for Tropical Dermatology.

Dr. Convit's scientific legacy includes not only his outstanding achievements in Tropical Medicine, chemotherapy, and immunotherapy of infectious diseases, but also his contribution to the careers of many others. He trained an important number of doctoral students, postdoctoral fellows, and undergraduate researchers who are now spread at academic research institutions around the world. In addition, he also trained generations of public health inspectors and physicians who now play an important role as health policy decision makers and spokespersons around the globe. His door was always open, and it was not a surprise to come into his house and see him having lunch with his patients.

One of Dr. Convit's great influences was on a group of young scientists who were keen to apply their skills in immunology and epidemiology to an important problem of developing countries. He reveled in learning about the new science and generously shared his vast clinical knowledge with everyone. This group became IMMLEP, the immunology of leprosy group at WHO, which was so successful at exchanging ideas, data, and reagents that it inspired the creation by WHO, United Nations Development Program (UNDP), and the World Bank of the Special Program for Research and Training in Tropical Diseases.

He had a great insight and understanding for people and treated everybody as equals, always with a mix of fondness and great respect. For his patients, he had a special gift for touching their hearts as well as their skin and conveying how much he cared about each of them. He was a humanitarian in every sense of the word.

Training under Dr. Convit's mentorship was with no doubt not only an educational, but an inspirational and exciting experience for those of us fortunate to work with him. He practiced what he taught as he worked until his last day of life. His research and mentorship activities are only a partial legacy of his extraordinary accomplishments. His true legacy lies in the role model he set as a husband, father, teacher, friend, and above all as a physician whose life revolved around the care of his patients. With his quiet, modest, and perseverant character, he will never be forgotten. We all feel a great sense of loss now that he is no longer with us. We have lost not only a great scientist, but a mentor and very dear friend.

